# Sex-specific and sex-independent steroid-related biomarkers in early second trimester maternal serum associated with autism

**DOI:** 10.1186/s13229-023-00562-5

**Published:** 2023-08-12

**Authors:** Deborah A. Bilder, Whitney Worsham, Scott Sullivan, M. Sean Esplin, Paul Burghardt, Alison Fraser, Amanda V. Bakian

**Affiliations:** 1 University of Utah Huntsman Mental Health Institute, 383 Colorow Drive, Room 360, Salt Lake City, UT 84108 USA; 2 University of Utah School of Medicine, Salt Lake City, UT USA; 3 Inova Health System, Falls Church, VA USA; 4 Intermountain Healthcare, Salt Lake City, UT USA; 5 Wayne State University, Detroit, MI USA; 6 University of Utah Huntsman Cancer Institute, Salt Lake City, UT USA

**Keywords:** Steroids, Sex hormone binding globulin, Estradiol, Metabolic syndrome, Developmental disability, Prenatal risk factors, Pregnancy

## Abstract

**Background:**

Prenatal exposure to maternal metabolic conditions associated with inflammation and steroid dysregulation has previously been linked to increased autism risk. Steroid-related maternal serum biomarkers have also provided insight into the in utero steroid environment for offspring who develop autism.

**Objective:**

This study examines the link between autism among offspring and early second trimester maternal steroid-related serum biomarkers from pregnancies enriched for prenatal metabolic syndrome (PNMS) exposure.

**Study design:**

Early second trimester maternal steroid-related serum biomarkers (i.e., estradiol, free testosterone, total testosterone, and sex hormone binding globulin) were compared between pregnancies corresponding to offspring with (*N* = 68) and without (*N* = 68) autism. Multiple logistic regression analyses were stratified by sex and gestational duration. One-way ANCOVA with post hoc tests was performed for groups defined by autism status and PNMS exposure.

**Results:**

Increased estradiol was significantly associated with autism only in males (AOR = 1.13 per 100 pg/ml, 95% CI 1.01–1.27, *p* = 0.036) and only term pregnancies (AOR = 1.17 per 100 pg/ml, 95% CI 1.04–1.32, *p* = 0.010). Autism status was significantly associated with decreased sex hormone binding globulin (AOR = 0.65 per 50 nmol/L, 95% CI 0.55–0.78, *p* < 0.001) overall and when stratified by sex and term pregnancy status. The inverse association between sex hormone binding globulin and autism was independent of PNMS exposure.

**Limitations:**

The relative racial and ethnic homogeneity of Utah’s population limits the generalizability of study results. Although significant differences by autism status were identified in concentrations of sex hormone binding globulin overall and of estradiol in participant subgroups, differences by PNMS exposure failed to reach statistical significance, which may reflect insufficient statistical power.

**Conclusion:**

Both elevated maternal serum estradiol in males only and low maternal serum sex hormone binding globulin in both sexes are associated with increased autism risk. Further investigation is merited to identify how steroid, metabolic, and inflammatory processes can interact to influence neurodevelopment in early second trimester.

## Background

Autism is a neurodevelopmental condition affecting approximately 2% of the population [1]. Autism is characterized by difficulties with social functioning, repetitive behaviors, restricted interests, insistence on sameness, and atypical sensory response [2]. Several pre-/perinatal autism risk factors associated with metabolic/steroid dysregulation and inflammation have been identified. Among these, maternal conditions are pre-existing/gestational diabetes and hypertension/pre-eclampsia (collectively hereafter referred to as prenatal metabolic syndrome, PNMS), increased pre-pregnancy body mass index (BMI), elevated LDL cholesterol, and gestational weight gain [3–12].

Changes in maternal metabolism and immune responses foster the health and growth of the fetus [13, 14]. Maternal hyperinsulinemia promotes transfer of nutritional resources to the fetus via the placenta [15, 16]. Maternal hypercortisolemia influences fetal response to stress via hypothalamic–pituitary–adrenal (HPA) programming and contributes to the immune privileged state in utero [14, 17]. The most vulnerable gestational period for disruptions within the maternofetoplacental unit to influence autism risk has yet to be determined, though some studies found an association between increased mid-gestation maternal stress and autism risk [18, 19]. The fetal HPA axis matures during this interval, and its programming can be influenced by maternal metabolic conditions and in utero stress [20–23]. Prior biomarker studies indicate greater autism risk associated with steroid dysregulation and inflammation during this gestational window [24–30].

In a pilot study with autistic and comparison groups enriched for PNMS exposure, Bilder et al [30] found higher estradiol and lower dihydroepiandrosterone (DHEA) levels in early second trimester maternal serum from term pregnancies associated with the presence of autism among offspring [30]. In a study of males with autism without consideration of PNMS exposure, Baron-Cohen et al [31] also identified higher estrogen levels (along with increased progesterone) in second trimester amniotic fluid [31]. Bilder et al [30] and Baron-Cohen et al [31] both interpreted their respective estrogen findings as suggestive of increased fetal steroidogenic activity in autism’s prenatal etiologic pathway. Additionally, Bilder et al [30] found lower sex hormone binding globulin (SHBG) levels in pregnancies associated with autism. As SHBG inactivates estradiol through binding [32], Bilder et al [30] proposed that this finding could potentiate the impact of high estradiol levels. Increased placental estradiol activity accelerates fetal HPA axis maturation during this gestational window [33], which may be relevant to autism pathogenesis. Maternal serum SHBG levels are also closely linked to PNMS as a predictor of gestational diabetes and pre-eclampsia [34–42]. How these prenatal autism biomarkers are influenced by fetal sex and pregnancy duration, both well-recognized autism risk factors, has yet to be investigated.

The current study extends the investigation of autism likelihood associated with early second trimester sex steroid-related serum biomarkers by enlarging autistic and non-affected offspring groups and including preterm pregnancies [30]. Both groups are enriched and matched for PNMS exposure. Study aims are to (1) evaluate autism risk associated with maternal serum SHBG and sex steroid hormone levels and (2) determine whether this relationship differs by fetal sex and term/preterm status.

## Methods

Approval for this study was obtained from the Utah Registry of Autism and Developmental Disabilities (URADD) Oversight Committee, the Utah State Office of Education, and Institutional Review Boards of the University of Utah (UU), Intermountain Healthcare (IM), Utah Department of Health and Human Services (UDHHS), and Resource for Genetic and Epidemiologic Research Review Committee. The latter is an oversight body that regulates Utah Population Database (UPDB) access. UPDB is a robust, comprehensive medical research resource that accesses many sets of high-quality, population-based, individual-level records [43].

### FASTER parent study

The First and Second Trimester Evaluation of Risk Study (FASTER) was an obstetrical study that ascertained over 12,000 women with singleton pregnancies living along Utah’s Wasatch Front from 1999 to 2002 [44]. Supplementary consent was obtained from 10,849 Utah FASTER participants for their residual serum samples to be used in additional research studies. These participants’ index offspring were identified through birth record linkage within the UPDB. This resulted in 3327 male and 3114 female offspring whose births coincided with their mothers’ FASTER participation.

### Autism status

In 2016, multiple data sources were linked within the UPDB to investigate autism birth risk factors. URADD was the primary source for autism status. URADD is administered through the UU Department of Psychiatry Huntsman Mental Health Institute with oversight from UDHHS and in cooperation with the Utah State Board of Education. URADD classifies the presence of autism using autism diagnostic billing codes and special education autism exceptionality status [45, 46]. Two FASTER birth years (2000, 2002) overlapped with URADD activities enhanced through Utah’s participation in the Centers for Disease Control and Prevention’s Autism and Developmental Disabilities Monitoring Network. This network uses record review methodology that has been validated in Utah [45, 47, 48]. Autism status was identified through diagnostic billing codes from UU and IM Enterprise Data Warehouses and statewide hospital discharge summaries. Among FASTER offspring linked to birth records, 168 were identified with autism.

### Prenatal metabolic syndrome (PNMS) exposure and covariates

Birth risk factors were obtained from birth records. PNMS exposure was defined as the presence of gestational hypertension, gestational diabetes, pre-/eclampsia, pre-existing diabetes (type 1 and 2), and/or pre-existing hypertension [30]. The prevalence of PNMS exposure among FASTER offspring approximated that of epidemiologic studies for these conditions [49, 50]. Pre-/perinatal characteristics previously associated with autism and/or PNMS were identified including child’s sex, parental ages, parental education, pre-pregnancy body mass index (BMI), pregnancy weight gain, birthweight, and gestational age. Term pregnancy was defined as ≥ 37 weeks gestation.

### Prenatal maternal serum

Maternal blood samples were collected at 15–18^6^ weeks gestation, between 1999 and 2002. Blood samples were centrifuged within 30 min, stored at 4 °C, and shipped overnight to a central laboratory for initial FASTER serum studies. Residual serum samples were frozen at − 80 °C. In 2017 and 2019, the first and second batch of samples, respectively, were shipped overnight on dry ice, stored at − 80 °C, thawed on wet ice, and aliquoted into pre-cooled tubes. In total, 2 thaw/refreeze cycles occurred prior to serum analyses.

### Sample selection

Steroid dysregulation occurs more frequently in pregnancies complicated by PNMS. Both autistic (44%) and comparison (47%) groups were enriched for PNMS exposure to examine sex steroid-related biomarkers across a stepwise change from the absence to presence of autism and PNMS: autism-/PNMS-, autism-/PNMS+ , autism+/PNMS- and autism+/PNMS+ . For the autism- group, 44 PNMS+ offspring were randomly selected and matched by sex and birth year to 44 PNMS- offspring. For the autism+ group, 31 PNMS + offspring (total identified) was matched to 45 PNMS- offspring by child’s sex and birth year. Corresponding early second trimester serum samples (*N* = 136) were located with the following group distributions: 36 autism-/PNMS-, 32 autism-/PNMS+ , 38 autism+ /PNMS-, and 30 autism+ /PNMS+ .

### Serum analysis

SHBG, estradiol, free testosterone, and total testosterone biomarker assays were performed in 96 well plates; plate loading occurred through an automated liquid handling system (Gilson Pipetmax). Commercially available ELISA kits were used per the manufacturer’s instructions, except where noted. Abcam (Boston, MA) ELISA kits measured estradiol, total testosterone, free testosterone; RayBiotech (Norcross, GA) ELISA kits measured SHBG. Estradiol required a 1:2 dilution in assay buffer to ensure samples fell within the dynamic range of the standard curve. Samples were tested in two batches with data from term offspring in the first batch reported in the pilot study [30]. Both term and preterm autism+ /PNMS+ samples (*n* = 30) were tested in the first batch along with term samples for autism-/PNMS- (*n* = 11), autism-/PNMS+ (*n* = 8), and autism+ /PNMS- (*n* = 28) samples. The samples in the second batch corresponded to both term and preterm pregnancies with the following autism and exposure status distribution: autism-/PNMS- (*n* = 25), autism-/PNMS+ (*n* = 24), and autism+ /PNMS- (*n* = 10).

### Statistical analysis

The distributions of biomarkers were examined for extreme outliers; one extreme total testosterone (7.47 pg/ml) and one free testosterone value (10.11 ng/ml) were observed and deleted. Distributions subsequently satisfied normality assumptions. Binary logistic regression models were fit to measure the association between autism and biomarkers. Two principal component factors (PCF) were extracted for the following sets of highly correlated covariates: gestational age and birthweight (PCF 1) and parental ages and education durations (PCF 2). Crude (unadjusted) models were initially formulated, and an adjusted model was fit by incorporating PCAs and additional covariates (i.e., sex, weight gain, and BMI). Two subsequent model sets were formulated stratifying by term/preterm status (PCF 1 was replaced with birthweight) and sex (sex was removed as a covariate).

For biomarkers which demonstrated significant associations with autism in the overall crude and adjusted models, one-way ANOVA and ANCOVA models were formulated to quantify the association between biomarker concentrations and a four-level measure of autism/PNMS exposure (i.e., autism-/PNMS-, autism-/PNMS+ , autism+/PNMS-, and autism+/PNMS+). The one-way ANOVA was initially formulated, and the ANCOVA model was subsequently fitted by incorporating PCF 1, PCF 2, and covariates above. Post hoc tests to control for multiple comparisons used the Sidak method [51].

Post hoc analyses using Pearson’s correlation and crude and adjusted linear regression, overall and stratified by autism status, were conducted to evaluate associations between SHBG and BMI. Because of the established inverse relationship between SHBG and obesity outside of pregnancy, these analyses explored how BMI may influence the relationship between autism risk and SHBG concentrations during pregnancy [42, 52, 53]. Covariates included in the adjusted model were sex, weight gain, PCF 1, and PCF 2. A sensitivity analysis was subsequently performed that included as an additional covariate a principal component factor (PCF 3) extracted for the highly correlated sex hormone levels estradiol, total testosterone, and free testosterone.

Analyses were conducted in SPSS v.28 and R (R Core Team 2021) with figures produced using ggplot2 [54]. An alpha of 0.05 was selected to assess statistical significance.

## Results

### Offspring characteristics

Serum analyses were conducted on 136 offspring with and without autism (*n* = 68, 73.5% male, 44.1% with PNMS exposure, 82.4% term; *n* = 68, 47.1% male, 47.1% with PNMS exposure, 60.3% term, respectively). See Table 1.

**Table 1:** Participant characteristics

Characteristics	Autistic group (*n* = 68)		Comparison group (*n* = 68)	
	*N*	%	*N*	%
Male	50	73.5	32	47.1
PNMS Exposure	30	44.1	32	47.1
Diabetes	14	20.6	< 11^a^	< 16.2^a^
Hypertension	22	32.4	23	33.8

	Mean (SD)	Range	Mean (SD)	Range
Maternal age (y)	29.1 (6.1)	19 to 45	28.4 (5.3)	16–43
Maternal education (y)	14.0 (1.9)	9 to 17	14.1 (2.3)	6–17
Paternal age (y)	31.0 (7.6)	20 to 52	29.9 (5.3)	19–43
Paternal education (y)	13.9 (1.9)	10 to 17	14.2 (2.2)	9–17
Gestational age^b^ (wk)	37.9 (2.6)	25 to 42	36.5 (2.3)	27–41
Birth weight (g)	3186 (712)	910 to 4590	2900 (641)	690–4082
Pre-pregnancy BMI	26.4 (6.8)	18.2 to 55.1	25.2 (4.1)	16.9–41.2
Pregnancy weight gain (lbs)	30.7 (14.3)	(− 3) to 62	28.3 (11.6)	4–60

^a^The specific number for cell counts of 10 or less is suppressed per Utah Population Database protocol

^b^In subsequent stratified analyses, the preterm cohort was born before 37 weeks gestation, and the term cohort was born 37 + weeks gestation

### SHBG and ASD risk

In crude and adjusted logistic regression analyses, autism status was significantly associated with decreased SHBG levels (OR = 0.66 per 50 nmol/L, 95% CI 0.56–0.77, *p* < 0.001; AOR = 0.65 per 50 nmol/L, 95% CI 0.55–0.78, *p* < 0.001, respectively). Similar results occurred when stratified by sex and gestational duration (see Table 2).

**Table 2:** Association between maternal serum sex hormone binding globulin levels and odds of offspring developing autism

Cohort	Mean	Range	SD	Crude models^a^			Adjusted models^a,b^		
				OR	95% CI	*P* Value	AOR	95% CI	*P* Value
Overall (*N* = 136)	316.42	57.05–1110.11	223.16	0.66	0.56–0.77	< 0.001	0.65	0.55–0.78	< 0.001
*By sex*									
Males (*n* = 82)	289.54	66.42–1110.11	204.93	0.69	0.58–0.83	< 0.001	0.7	0.57–0.85	< 0.001
Females (*n* = 54)	357.23	57.05–1030.56	244.65	0.54	0.37–0.79	0.001	0.58	0.35–0.95	0.031
*By gestational age category*^*c*^									
Term (*n* = 97)	274.95	57.05–1110.11	224.62	0.67	0.55–0.82	< 0.001	0.67	0.53–0.85	< 0.001
Preterm (*n* = 39)	419.56	77.69–813.79	184.81	0.63	0.45–0.86	0.004	0.5	0.30–0.85	0.01

^a^OR and AOR are calculated for every 50 nmol/L increase in sex hormone binding globulin

^b^Adjusted for Principal Component Factor 1 (gestational age, birthweight), Principal Component Factor 2 (maternal age, paternal age, maternal education duration, paternal education duration), pre-pregnancy body mass index (BMI), gestational weight gain, newborn sex. When stratified by sex, sex was removed as a covariate. When stratified by gestational age category, principal component factor 1 was replaced with birthweight

^c^Preterm < 37 weeks gestation; term ≥ 37 weeks gestation

### Estradiol, free testosterone, total testosterone, and ASD risk

In the crude logistic regression analysis, autism was significantly associated with increased estradiol levels (OR = 1.09 per 100 pg/ml, 95% CI 1.01–1.17, *p* = 0.02); this relationship did not reach significance in the adjusted model (AOR = 1.08 per 100 pg/ml, 95% CI 1.00–1.17, *p* = 0.07). When stratified by gestational duration, crude and adjusted models for term pregnancies (but not preterm) demonstrated significant associations between estradiol levels and autism (OR = 1.14 per 100 pg/ml, 95% CI 1.03–1.26, *p* = 0.01; AOR = 1.17 per 100 pg/ml, 95% CI 1.04–1.32, *p* = 0.01, respectively). When stratified by sex, the association between estradiol and autism was only significant in males after adjusting for covariates (AOR = 1.13 per 100 pg/ml, 95% CI 1.01–1.27, *p* = 0.04) (See Table 3). All analyses for free and total testosterone found no significant associations with autism (See Tables 4 and 5).

**Table 3:** Association between maternal serum estradiol levels and odds of offspring developing autism

Cohort	Mean	Range	SD	Crude models^a^			Adjusted models^a,b^		
				OR	95% CI	*P* Value	AOR	95% CI	*P* Value
Overall (*N* = 135)	1096.01	192.50–2524.54	500.66	1.09	1.01–1.17	0.023	1.08	1.00–1.17	0.065
*By Sex*									
Males (*n* = 81)	1143.94	330.83–2285.98	478.24	1.09	0.99–1.21	0.083	1.13	1.01–1.27	0.036
Females (*n* = 54)	1024.11	192.50–2524.54	528.9	1.06	0.95–1.18	0.294	0.94	0.79–1.11	0.438
*By gestational age category*^*c*^									
Term (*n* = 96)	1153.46	330.83–2285.98	444.61	1.14	1.03–1.26	0.011	1.17	1.04–1.32	0.01
Preterm (*n* = 39)	954.59	192.50–2524.54	600.7	0.99	0.89–1.11	0.82	0.97	0.85–1.11	0.687

^a^OR and AOR are calculated for every 100 pg/ml increase in estradiol

^b^Adjusted for Principal Component Factor 1 (gestational age, birthweight), Principal Component Factor 2 (maternal age, paternal age, maternal education duration, paternal education duration), pre-pregnancy body mass index (BMI), gestational weight gain, newborn sex. When stratified by sex, sex was removed as a covariate. When stratified by gestational age category, principal component factor 1 was replaced with birthweight

^c^Preterm < 37 weeks gestation; term ≥ 37 weeks gestation

**Table 4:** Association between total testosterone and odds of offspring developing autism

Cohort	Mean	Range	SD	Crude models^a^			Adjusted models^a,b^		
				OR	95% CI	*P* Value	AOR	95% CI	*P* Value
Overall (*N* = 135)	0.9	0.22–3.50	0.47	0.79	0.38–1.64	0.52	1.01	0.41–2.50	0.979
*By sex*									
Males (*n* = 81)	0.9	0.34–3.50	0.5	0.6	0.24–1.51	0.275	1.02	0.35–2.97	0.967
Females (*n* = 54)	0.89	0.22–2.10	0.42	1.34	0.35–5.10	0.672	0.36	0.05–2.45	0.293
*By gestational age category*^c^									
Term (*n* = 97)	0.87	0.30–2.31	0.39	1.05	0.38–2.95	0.921	1.21	0.34–4.30	0.768
Preterm (*n* = 38)	0.96	0.22–3.50	0.62	0.6	0.15–2.35	0.466	0.43	0.10–1.87	0.262

^a^OR and AOR are calculated for every 1 ng/ml increase in total testosterone

^b^Adjusted for Principal Component Factor 1 (gestational age, birthweight), Principal Component Factor 2 (maternal age, paternal age, maternal education duration, paternal education duration), pre-pregnancy body mass index (BMI), gestational weight gain, newborn sex. When stratified by sex, sex was removed as a covariate. When stratified by gestational age category, principal component factor 1 was replaced with birthweight

^c^Preterm < 37 weeks gestation; term ≥ 37 weeks gestation

**Table 5:** Association between free testosterone and odds of offspring developing autism

Cohort	Mean	Range	SD	Crude models^a^			Adjusted models^a,b^		
				OR	95% CI	*P* Value	AOR	95% CI	*P* Value
Overall (*N* = 134)	1.27	0.00–4.91	0.88	1.01	0.68–1.48	0.98	1.08	0.68–1.71	0.74
*By sex*									
Males (*n* = 80)	1.31	0.00–4.91	0.95	0.84	0.53–1.36	0.483	1.05	0.62–1.79	0.846
Females (*n* = 54)	1.20	0.04–3.26	0.76	1.35	0.64–2.84	0.434	0.53	0.18–1.62	0.265
*By gestational age category*^*c*^									
Term (*n* = 96)	1.26	0.06–4.02	0.78	0.96	0.57–1.63	0.888	0.96	0.51–1.83	0.906
Preterm (*n* = 38)	1.27	0.00–4.91	1.1	1.08	0.58–2.03	0.809	1.01	0.49–2.08	0.981

^a^OR and AOR are calculated for every 1 pg/ml increase in free testosterone

^b^Adjusted for Principal Component Factor 1 (gestational age, birthweight), Principal Component Factor 2 (maternal age, paternal age, maternal education duration, paternal education duration), pre-pregnancy body mass index (BMI), gestational weight gain, newborn sex. When stratified by sex, sex was removed as a covariate. When stratified by gestational age category, principal component factor 1 was replaced with birthweight

^c^Preterm < 37 weeks gestation; term ≥ 37 weeks gestation

### Effects of PNMS exposure on ASD risk associated with SHBG

**Fig. 1:**
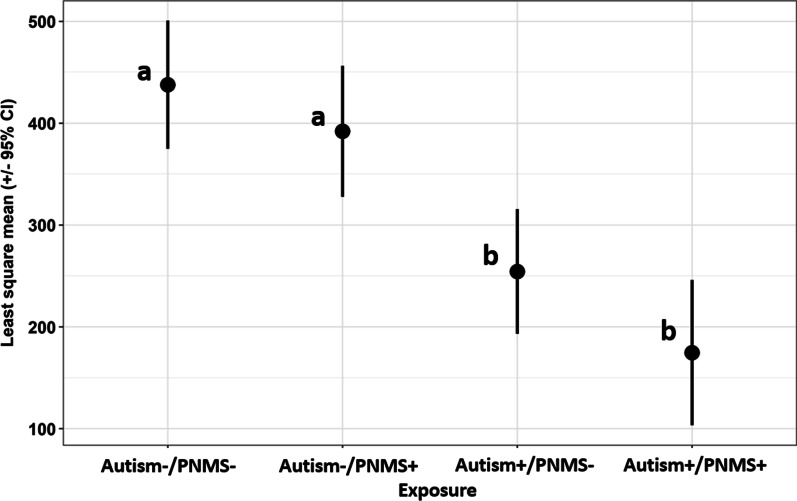
Sex Hormone Binding Globulin (SHBG) levels by autism and Prenatal Metabolic Syndrome (PNMS) exposure status. ANCOVA included covariates sex, BMI, gestational weight gain, principal component factor 1 (birthweight and gestational age) and principal component factor 2 (parental ages and durations of education), with Sidak correction for multiple comparisons. Values that do not share a letter differ significantly from each other.

Table 6 provides a description of offspring characteristics stratified into four groups based on autism and PNMS exposure that were used in the ANOVA and ANCOVA models. Both ANOVA and ANCOVA models yielded significant overall effects for autism/PNMS exposure on mean SHBG levels (*F*(3135) = 20.0 *p* < 0.001, *F*(8131) = 10.4 *p* < 0.001; (*F*(3131) = 12.3, *p* < 0.001). Figure 1 depicts the results of the post hoc tests which indicated that statistically significant differences remained in the least square means of SHBG concentrations between autism+/PNMS+ and autism-/PNMS- groups (mean difference = − 263.00, 95% CI = − 395.83 − − 130.18, *p* < 0.001), autism+/PNMS+ and autism-/PNMS+ groups (mean difference = − 217.37, 95% CI = − 347.63 − − 87.10, *p* < 0.001), autism+/PNMS- and autism-/PNMS- groups (mean difference = − 183.36, 95% CI = − 300.64 − − 66.08, *p* < 0.001), and autism+/PNMS- and autism-/PNMS+groups (mean difference = − 137.72, 95% CI = − 260.77 − − 14.67, *p* = 0.02).

**Table 6:** Participant characteristics by autism and prenatal metabolic syndrome exposure (PNMS) classification

	Autism-/PNMS- (*n* = 36)		Autism-/PNMS + (*n* = 32)		Autism + /PNMS- (*n* = 38)		Autism + /PNMS + (*n* = 30)	
	*N*	%	*N*	%	*N*	%	*N*	%
Male	17	47.2	15	46.9	28	73.7	22	73.3
Diabetes	N/A	N/A	14	43.8	N/A	N/A	< 11^a^	< 36.6^a^
Hypertension	N/A	N/A	22	68.8	N/A	N/A	23	76.7

	Mean (SD)	Range	Mean (SD)	Range	Mean (SD)	Range	Mean (SD)	Range
Maternal age (y)	28.6 (5.5)	16 to 38	28.1 (5.2)	18 to 43	29.0 (6.4)	19 to 42	29.2 (5.8)	21 to 45
Maternal education (y)	13.9 (2.7)	6 to 17	14.4 (1.7)	11 to 17	13.9 (2.1)	9 to 17	14.1 (1.7)	11 to 17
Paternal age (y)	30.2 (5.0)	19 to 40	29.5 (5.7)	20 to 43	32.3 (7.9)	21 to 52	29.3 (7.1)	20 to 52
Paternal education (y)	14.3 (2.5)	9 to 17	14.1 (1.9)	12 to 17	14.0 (2.0)	10 to 17	13.8 (1.8)	10 to 17
Gestational age^b^ (wk)	36.9 (1.9)	30 to 41	36.2 (2.6)	27 to 40	38.2 (2.9)	25 to 42	37.5 (2.1)	31 to 40
Birth weight (g)	2942 (546)	1550 to 4082	2851 (739)	690 to 4054	3213 (733)	910 to 4443	3153 (696)	1332 to 4590
Pre-pregnancy BMI	24.6 (3.8)	16.9 to 32.8	25.9 (4.4)	18.5 to 41.2	24.9 (6.6)	18.2 to 55.1	28.5 (6.7)	20.0 to 43.6
Pregnancy Weight gain (lbs)	26.6 (10.3)	4 to 50	30.3 (12.8)	8 to 60	28.1 (11.7)	2 to 47	34.2 (16.8)	− 3 to 62

^a^The specific number for cell counts of 10 or less is suppressed per Utah Population Database protocol

^b^In stratified analyses, the preterm cohort was born before 37 weeks gestation, and the term cohort was born 37 + weeks gestation

### Association between SHBG and BMI

**Fig. 2:**
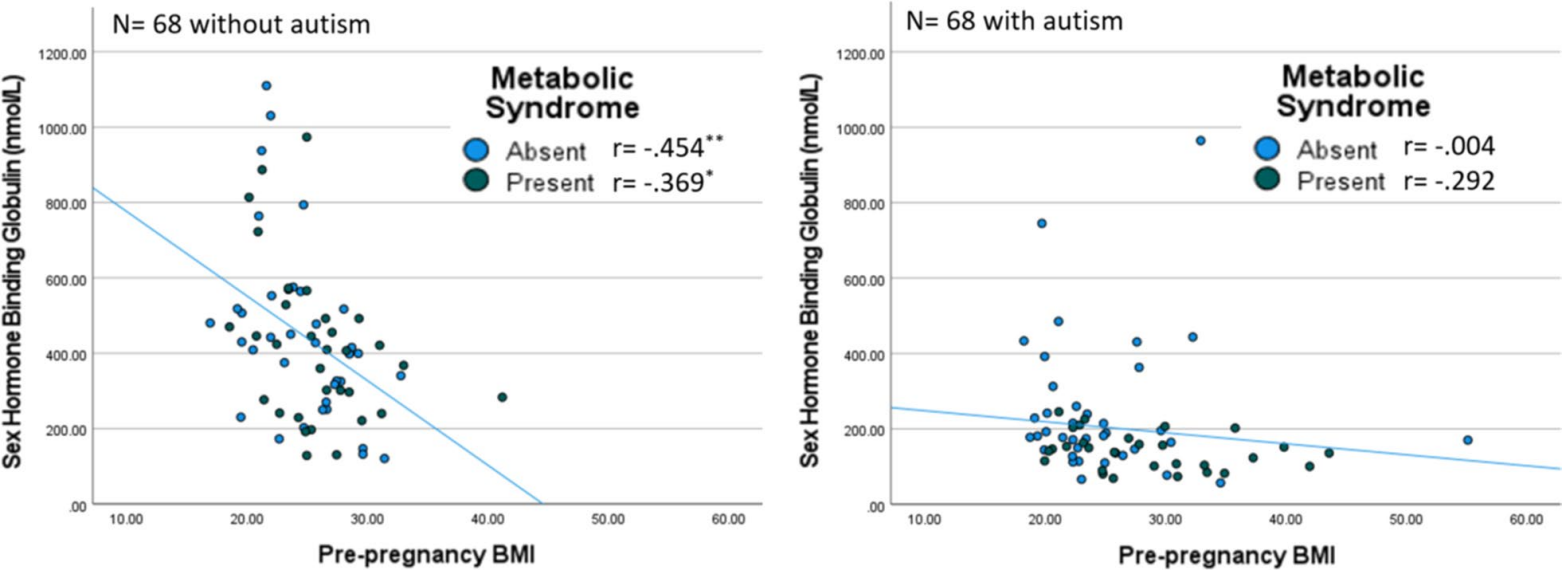
Scatterplot between maternal serum sex hormone binding globulin levels and pre-pregnancy body mass index (BMI) by prenatal metabolic syndrome exposure and autism among offspring.

In post hoc analyses, BMI demonstrated a statistically significant inverse relationship with SHBG levels in crude and adjusted models for the overall cohort (*β* = − 10.35, *p* = 0.002; *β* = − 12.02, *p* < 0.001, respectively) and among those without autism (*β* = − 22.45, *p* < 0.001; *β* = − 24.22, *p* < 0.001, respectively). However, no associations were found between maternal serum SHBG levels and BMI for offspring with autism in crude or adjusted models (*β* = − 2.92, *p* = 0.28; *β* = − 4.29, *p* = 0.14, respectively). The lack of association between SHBG levels and BMI in the autism group was particularly notable in the presence of PNMS exposure. The sensitivity analysis adding PCF 3 to the adjusted models resulted in comparable findings. See Fig. 2 and Table 7.

**Table 7:** Association between sex hormone binding globulin and Body Mass Index (BMI) by autism status

Cohort	Crude models			Adjusted models^a^			Sensitivity Analyses^b^		
	β	95% CI	*P* Value	β	95% CI	*P* Value	β	95% CI	*P* Value
Overall	− 10.35	− 16.90–− 3.80	0.002	− 12.02	− 18.38–− 5.66	< 0.001	− 12.02	− 18.41–− 5.62	< 0.001
*Autism status*									
Present	− 2.92	− 8.33–2.48	0.284	− 4.29	− 10.01–1.42	0.138	− 4.23	− 9.99–1.53	0.147
Absent	− 22.46	− 34.52–− 10.41	< 0.001	− 24.22	− 36.11–− 12.33	< 0.001	− 24.46	− 36.82–− 12.10	< 0.001

^a^Adjusted for Principal Component Factor 1 (PCF 1: gestational age, birthweight), Principal Component Factor 2 (PCF 2: maternal age, paternal age, maternal education duration, paternal education duration), gestational weight gain, newborn sex

^b^Adjusted for PCF 1, PCF 2, gestational weight gain, newborn sex, and Principal Component Factor 3 (PCF 3: total testosterone, free testosterone, and estradiol levels)

## Discussion

Likelihood of autism among offspring was associated with low SHBG and increased estradiol levels in early second trimester maternal serum. SHBG levels demonstrated a significant inverse association with the presence of autism among offspring overall and across gestational age and sex categories. SHBG production occurs in the liver and is influenced by insulin sensitivity and body fat composition during pregnancy [36, 52, 55]. Although SHBG’s main function is to bind sex steroids to reduce free sex hormone levels, SHBG has also been found to link inversely to insulin resistance independent of sex hormone levels [56]. Wallace et al [56] provide a review of observational and genetic studies that describe the relationship between low SHBG levels and type 2 diabetes mellitus and support insulin resistance as a mechanistic link underlying this association. SHBG has previously been used as a proxy for gestational insulin sensitivity [41] as estradiol and testosterone play a limited role in SHBG regulation during pregnancy [35]. Current study findings support this prior work as the relationship between SHBG and autism likelihood was unchanged following adjustment for sex hormone levels. Prenatal maternal serum SHBG levels have previously been found to correlate negatively with fasting plasma glucose, insulin, and C-peptide levels [34, 35]. Serum SHBG levels are lower in women with gestational diabetes [36] and have demonstrated predictive value for gestational diabetes and hypertension [34, 35, 37–42]. Interestingly, the current study found lower SHBG levels in the autism cohorts with and without PNMS exposure. If lower maternal serum SHBG were to reflect insulin resistance, study findings suggest that insulin insensitivity could be occurring in the early second trimester of pregnancies associated with autism even in the absence of subsequent clinical PNMS manifestations.

BMI is recognized as a major determinant of SHBG levels and has been inversely associated with SHBG in prior obstetrical studies [42, 52, 53]. The connection between BMI and SHBG levels has been attributed to higher liver fat content because lipogenesis suppresses hepatic SHBG synthesis [52, 55, 57]. While the post hoc analysis of SHBG and BMI demonstrated a significant association in the overall cohort, this relationship appeared to be driven entirely by the unaffected cohort and was absent in pregnancies from which offspring developed autism. Instead, SHBG levels in the autism group were low across the BMI range. Low SHBG levels in the autism group regardless of BMI suggest that metabolic processes could be occurring during pregnancies associated with autism that supersede SHBG synthesis suppression by hepatic lipogenesis.

A pilot version of the current study found higher maternal serum estradiol levels, in combination with lower DHEA and SHBG levels, to be associated with increased likelihood of autism among term offspring [30]. Collectively, these findings were interpreted as indicating increased steroidogenic activity by the fetus at risk for autism during early second trimester, as DHEA from both maternal and fetal adrenal glands acts as a substrate for placental estradiol synthesis [58, 59]. Additionally, higher placental estradiol activity during this gestational window can accelerate fetal HPA axis maturation [33, 60]. By extending the pilot study with 65 additional participants, 39 of whom were born preterm, current findings indicate that the link between increased maternal serum estradiol and autism likelihood appears specific to male sex and term delivery. In an exclusively male autism cohort, Baron-Cohen et al [31] identified elevated mid-gestation amniotic fluid estrogen levels and attributed this finding to their longstanding theory that autism is caused by the impact of increased fetal steroidogenic activity on sex-specific brain development. Amniotic fluid and maternal serum estradiol levels reflect complementary components of the maternofetoplacental unit as amniotic fluid at this stage in pregnancy is populated by fetal urine output, while maternal serum estradiol is produced primarily by the placenta using fetal and maternal substrates [58, 59, 61]. Further study is needed to understand whether the link between higher amniotic fluid steroid levels, and autism is also limited to male and term offspring.

Unlike the current study’s null findings between estradiol and autism likelihood in the overall model following adjustment for covariates, Windham et al [28] found lower levels of another estrogen (i.e., estriol) in mid-gestation maternal serum significantly linked to autism. Study design differences may account for these discrepancies as the Windham et al [28] study used a diverse, population-based sample rather than a small sample enriched for PNMS exposure; estradiol and estriol also differ significantly in their receptor binding capacity and clearance rate within maternal serum. Further investigation into steroid-related maternal serum biomarkers within a large population-based sample is merited to understand how the mother, pregnancy, and child’s characteristics could influence the relationship between maternal mid-gestation serum estrogen levels and autism.

The sex-specific association between maternal serum estradiol and the likelihood of autism among offspring may reflect sex differences in fetal response to prenatal adversity that place male newborns at greater risk for complications [62–65]. Estradiol has a bimodal effect on inflammation with lower levels enhancing T-cell and pro-inflammatory cytokine responses and higher levels (i.e., during pregnancy) reducing these immune responses while promoting B-cell antibody production [66]. Subsequently, cell-mediated autoimmune diseases (e.g. rheumatoid arthritis) improve during pregnancy while autoantibody-mediated conditions worsen (e.g., systemic lupus erythematosus). Maternal anti-fetal brain autoantibodies have been implicated in autism, and mouse models demonstrate that anti-fetal brain antibodies produce behavior changes (e.g., increased repetitive behaviors, reduced social interest) in males only [67, 68]. If extended exposure to high estradiol levels influences inflammatory processes, then, affected pregnancies carried to term may increase offspring’s susceptibility to subsequent adverse effects. Interestingly, Windham et al. also suggested that the inverse relationship they identified between mid-gestation maternal serum estrogen levels and autism likelihood could be attributed to hormonal influences on the immune system [28].

While outside the context of pregnancy, SHBG has also been directly linked to inflammation. SHBG has been inversely associated with the inflammatory marker C-reactive protein and reduces inflammatory processes in vitro [69, 70]. Introducing SHBG to adipocytes and macrophages suppresses the pro-inflammatory response induced by lipopolysaccharide [70]. If these properties extend to pregnancy, low SHBG levels could herald a pro-inflammatory state in the maternal compartment that could adversely affect fetal development. Prior maternal serum biomarker studies have demonstrated higher autism likelihood associated with inflammation during the gestational window in which current study samples were collected [24–27].

This study harnessed a unique collection of resources available in Utah to investigate maternal metabolic conditions and the in utero steroid environment associated with ASD. Because serum SHBG levels do not require timed sample collection, a proxy for insulin sensitivity can be evaluated within banked serum from an obstetrical cohort large enough to examine risk associated with a childhood outcome affecting 2% of the population [36, 41]. Study findings justify investigation into early second trimester phenomena that may suppress maternal SHBG production and influence fetal neurodevelopment even in the absence of subsequent clinical PNMS manifestations.

## Limitations

The relative racial and ethnic homogeneity of Utah’s population limits the generalizability of study results. Although significant differences by autism status were identified in concentrations of SHBG overall and of estradiol in participant subgroups, differences by PNMS exposure failed to reach statistical significance, which may reflect insufficient statistical power. To eliminate potential batch effects on the comparison between preterm and term autism+/PNMS + offspring, all autism+/PNMS+ offspring were analyzed within the first batch which limits the interpretation of results related to PNMS exposure between the autism subgroups across batches. Compared to steroid hormones, inflammatory serum biomarkers are less stable over time and across freeze–thaw cycles [71, 72 . The absence of accompanying inflammatory biomarkers also limits this study’s ability to inform how findings relate to co-occurring inflammatory processes. Further work is needed to understand how lower SHBG and elevated estradiol levels may coincide with in utero processes that could collectively contribute to autism’s development.

## Conclusion

Study findings suggest that maternal SHBG production is suppressed during the early second trimester of pregnancies associated with autism among offspring independent of fetal sex, gestational duration, BMI, sex hormone levels, and subsequent PNMS emergence. Autism likelihood associated with higher maternal estradiol levels appears limited to male offspring and term birth. Understanding how prenatal maternal SHBG and estradiol levels are linked to autism among offspring could facilitate early detection screening strategies and foster the development of therapeutic interventions to optimize fetal health during this critical period of neurodevelopment.

## Data Availability

The data that support the findings of this study are not openly available due to the sensitivity of human data collected by the Utah Registry of Autism and Developmental Disabilities and the Utah Population Database. Access to this data requires approval from the University of Utah, the Utah Department of Health and Human Services, the Utah Population Database, Intermountain Healthcare, and the Utah State Board of Education.

## Abbreviations

HPA: Hypothalamic–pituitary–adrenal
PNMS: Prenatal metabolic syndrome
DHEA: Dehydroepiandrosterone
URADD: Utah Registry of Autism and Developmental Disabilities
UDHHS: Utah Department of Health and Human Services
UU: University of Utah
UPDB: Utah Population Database
FASTER: First and Second Trimester Evaluation of Risk
BMI: Body mass index
DHEAS: Dehydroepiandrosterone sulfate
SHBG: Sex hormone binding globulin
PCF: Principal component factor
ANOVA: Analysis of variance
ANCOVA: Analysis of covariance
